# Premature mortality attributable to socioeconomic inequality in England between 2003 and 2018: an observational study

**DOI:** 10.1016/S2468-2667(19)30219-1

**Published:** 2019-12-05

**Authors:** Dan Lewer, Wikum Jayatunga, Robert W Aldridge, Chantal Edge, Michael Marmot, Alistair Story, Andrew Hayward

**Affiliations:** a UCL Collaborative Centre for Inclusion Health, University College London, London, UK; b Institute for Health Informatics, University College London, London, UK; c Institute of Epidemiology and Health Care, University College London, London, UK; d Find and Treat, University College London Hospitals, London, UK

## Abstract

**Background:**

Low socioeconomic position is consistently associated with increased risk of premature death. The aim of this study is to measure the aggregate scale of inequality in premature mortality for the whole population of England.

**Methods:**

We used mortality records from the UK Office for National Statistics to study all 2 465 285 premature deaths (defined as those before age 75 years) in England between Jan 1, 2003, and Dec 31, 2018. Socioeconomic position was defined using deciles of the Index of Multiple Deprivation: a measure of neighbourhood income, employment, education levels, crime, health, availability of services, and local environment. We calculated the number of expected deaths by applying mortality in the least deprived decile to other deciles, within the strata of age, sex, and time. The mortality attributable to socioeconomic inequality was defined as the difference between the observed and expected deaths. We also used life table modelling to estimate years-of-life lost attributable to socioeconomic inequality.

**Findings:**

35·6% (95% CI 35·3–35·9) of premature deaths were attributable to socioeconomic inequality, equating to 877 082 deaths, or one every 10 min. The biggest contributors were ischaemic heart disease (152 171 excess deaths), respiratory cancers (111 083) and chronic obstructive pulmonary disease (83 593). The most unequal causes of death were tuberculosis, opioid use, HIV, psychoactive drugs use, viral hepatitis, and obesity, each with more than two-thirds attributable to inequality. Inequality was greater among men and peaked in early childhood and at age 40–49 years. The proportion of deaths attributable to inequality increased during the study period, particularly for women, because mortality rates among the most deprived women (excluding cardiovascular diseases) plateaued, and for some diseases increased. A mean of 14·4 months of life before age 75 years are lost due to socioeconomic inequality.

**Interpretation:**

One in three premature deaths are attributable to socioeconomic inequality, making this our most important public health challenge. Interventions that address upstream determinants of health should be prioritised.

**Funding:**

National Institute of Health Research; Wellcome Trust.

## Introduction

People with low socioeconomic position, defined by their job, qualifications, income, wealth, or where they live, are more likely to die young than people with a high socioeconomic position. Short life expectancy among poor industrial workers was documented in the nineteenth century and since then socioeconomic inequalities in mortality have been observed worldwide.^1^, ^2^ Reducing this inequality is now a central health policy objective but progress has been limited.^3^, ^4^ Studies of changes in inequality over recent decades have found a persistence or widening in the difference between rich and poor.^5^, ^6^, ^7^ This disparity represents a failure in the international response to health inequalities.

Epidemiologists and demographers have developed various methods of measuring health inequality.^8^ Differences in life expectancy between the richest and poorest (using the Slope Index of Inequality) is one approach, and in England in 2018, this was 9·3 years for men and 7·3 years for women.^9^ Another common measure is the standardised mortality ratio, which has been used to show, for example, that people working in occupations such as labourers and cleaners have double the suicide risk of the general population.^10^ Most measures provide an estimate of the individual-level risk associated with low socioeconomic position and therefore do not convey the aggregate loss of life associated with inequality. Some studies have used population attributable fractions to estimate the proportion of deaths that would be avoided if everyone had the same mortality risk as the least deprived group. These studies provide insight into the aggregate effect of inequality, but typically use single markers of socioeconomic position, such as occupation and education, limiting their policy implications.^11^, ^12^ They are also based on defined cohorts rather than whole population data, and are therefore subject to selection bias and mean that the total numbers of deaths attributable to inequality cannot be directly reported.

Simple measures are needed that communicate the scale of inequality to policy makers, politicians, and the public; monitor progress over time; and support targeted action by identifying diseases and population groups where inequality is greatest. We therefore aimed to show how the aggregate scale of socioeconomic inequality in mortality can be reported at a population level, using readily available mortality statistics, and to disaggregate inequality by demographic group, geographical area, and cause of death.

**Research in context**

**Evidence before this study**

Low socioeconomic position has been consistently identified as a predictor of premature mortality, but the aggregate scale of socioeconomic inequality on mortality is still unclear. We searched PubMed and Google Scholar from inception until Jan 21, 2018, for (“deprivation” OR “poverty” OR “income” OR “socioeconomic” OR “inequality”) AND (“attributable” OR “years of life” OR “YLL”) AND (“mortality” OR “death”*). An international meta-analysis of cohort studies reporting mortality risk associated with occupational status estimated that 19% of premature deaths among men and 15% among women could be avoided if occupational inequalities were eliminated. A review of longitudinal data in 13 European countries reported that between 10% and 60% of premature deaths would be avoided if all members of the study had the same mortality risk as those with the highest level of education. Six further studies in Belgium, Canada, the USA, and Australia reported that between 22% and 42% of premature deaths are attributable to area-based socioeconomic inequalities. In all studies, inequality was greater among men than among women. In most cases, inequality was defined by individual attributes such as education and occupation. Studies giving results within disease groups found the highest inequality for drug-use disorders, obstructive lung disease, lung cancer, suicide, and cirrhosis, and less inequality for cancers of the bowel and breast.

**Added value of this study**

We use two indicators of socioeconomic inequality: mortality attributable to inequality (referred to as MASI: the number and proportion of premature deaths that can be attributed to socioeconomic differences), and the years of life lost to socioeconomic inequality (the reduction in life expectancy before 75 years attributable to inequality). We applied these indicators to the whole population of England over the period 2003–18, allowing direct reporting of aggregate numbers of death and avoiding selection bias. Our study uses an index of inequality that combines data on income, employment, education levels, crime, availability of services, and the local environment in individuals' neighbourhoods, providing insight into health inequalities associated with upstream socioeconomic circumstances. We studied cause-specific inequality in much greater detail than in previous studies, including 156 causes of death. Our findings showed little or no inequality in some diseases such as cancers of the skin, blood, breast, eye, and brain, and for cystic fibrosis, whereas three-quarters of premature deaths caused by tuberculosis, HIV, and illicit drugs were attributable to socioeconomic inequality. We studied inequality in premature mortality by age, sex, and deprivation, showing that three-quarters of deaths among men aged 35–49 years in the poorest areas were attributable to inequality. Although mortality reduced over the study period, the proportion attributable to inequality increased, particularly for women. Inequalities were tempered by converging rates of cardiovascular mortality between deprivation groups, whereas inequalities in other diseases, including respiratory diseases among women, plateaued or even worsened.

**Implications of all the available evidence**

Inequalities in premature mortality are persistent despite reducing total mortality, and we estimate that one in three premature deaths can be attributed to socioeconomic position. Studies consistently show that absolute inequalities in mortality have reduced in the past two decades among men. Inequalities in premature mortality associated with so-called upstream factors, such as neighbourhood deprivation, are similar or greater than inequalities associated with behavioural factors, such as alcohol, tobacco, and physical activity. Local public health action and national policies need to prioritise interventions that address the determinants of these inequalities. Mortality attributable to inequality can be used to monitor inequalities in whole populations and subpopulations, in conjunction with existing measures of health inequality.

## Methods

### Data sources

We obtained individual-level data for all deaths at ages 0–74 years from the UK Office for National Statistics. Data included the decedent's age at death, sex, postcode of residence, and the underlying cause of death by International Classification of Diseases-10 code from Jan 1, 2003 and Dec 31, 2018. Postcodes were used to derive the decedent's neighbourhood, defined by Lower Layer Super Output Areas, which are small areas of around 1500 residents, of which there are 32 844 in England.^13^ Deprivation was defined by deciles of the Index of Multiple Deprivation 2015 (IMD).^14^ This measure combines information about each Lower Layer Super Output Area in seven domains: income, employment, education levels, crime, health, availability of services, and local environment. Each domain is based on a group of indicators. We recalculated IMD to exclude measures of health and mortality. Mid-year population estimates by lower super output area, sex, and single year of age from 2003 to 2017 are publicly available.^15^ At the time of publication, population estimates for 2018 were available by local authority, age, and sex, but not by IMD or Lower Layer Super Output Area. We therefore created a population estimate by increasing the age of the 2017 populations by 1 year, assuming the same number of newborn babies, controlling to the 2018 population estimates at local authority level.

### Statistical analysis

First, we stratified the mortality and population data according to 5 year age group (with infants younger than 1 year in a separate group), sex, deprivation decile, and 4 year period (2003–06, 2007–10, 2011–14, and 2015–18), yielding 1280 strata. Within each age group, sex, and period (128 strata), the mortality rate for the least deprived decile was used as a reference group and applied to the other deciles to produce a number of expected deaths. We calculated the mortality attributable to socioeconomic inequality (MASI) as the difference between the observed and expected deaths, expressed as both a percentage (equivalent to a population attributable fraction)^8^ and a number of excess deaths. 95% CIs were estimated using Monte-Carlo simulation, in which 10 000 values of the number of deaths in each stratum were sampled from a poisson distribution. We reported MASI by age, sex, and deprivation. In subnational analysis, we calculated MASI for 326 local authority districts using the same national reference rates.

Second, we constructed life tables for cohorts of 100 000 individuals using the method described by the Office for National Statistics,^16^ using mortality rates by single-year of age, with separate life tables for each sex and IMD decile (20 life tables). Years of life lost to inequality was calculated as the difference between the years lost due to death before age 75 years in each cohort and the corresponding least deprived cohort. We also expressed years of life lost as a percentage for comparison to MASI. The purpose of life table modelling was to give greater weight to deaths that occur at a younger age and to assess potential bias due to differing age structures in deprivation groups. We did not calculate years of life lost for subnational areas because mortality within strata at subnational level would be required, which might be unstable, while MASI only uses stratum-specific mortality at national level.

We repeated these procedures by underlying cause of death grouped using a three-level hierarchy. First, we grouped deaths by International Classification of Diseases-10 chapter (such as I00–I99; circulatory diseases), with chapters causing fewer than 1000 deaths assigned to Other. Second, if a chapter included any subcategories (such as I20–I25; ischaemic heart disease) responsible for more than 1000 deaths, we created a second level showing these subcategories. Finally, if the subcategory included any three-digit diagnoses (such as I20; acute myocardial infarction) responsible for more than 1000 deaths, we created a third level for these diagnoses. We also identified chapters or subcategories causing more than 50 000 premature deaths as major causes of death. After this grouping process, we separated neonatal deaths (all causes during the first 28 days of life).

As a sensitivity analysis, we calculated MASI using different quantiles of IMD, in which the least deprived quantile in each case (eg, the least deprived fifth of lower super output areas) was used as the reference group (appendix p 11).

For descriptive purposes, we reported standardised mortality rates by sex and time period using the European Standard Population 2013.^17^ As an exploratory analysis of changes over time (not prespecified), we reported the change in standardised mortality rates between the first (2003–06) and final period (2015–18), stratified by IMD decile, major cause of death, and sex (appendix pp 12, 13).

Analyses were done using R version 3.5.1. We have posted a version of the analysis code that only requires publicly available information. This project was approved by the UCL Research Ethics Committee (ref 13275/001) and by the Office for National Statistics Microdata Release Panel (ref 1010614).

### Role of the funding source

The funder of the study had no role in study design, data collection, data analysis, data interpretation, or writing of the report. The corresponding author had full access to all the data in the study and all authors shared final responsibility for the decision to submit for publication.

## Results

The study covered 780 million person-years in people aged younger than 75 years, with 2 465 285 premature deaths. During the study period premature mortality rates decreased for both men and women and in all deprivation groups. Reductions in absolute mortality rates were greater for more deprived groups, whereas relative reductions were greater for less deprived groups, leading to an increase in MASI (table 1).

**Table 1:** Inequality over time

		**2003–06**	**2007–10**	**2011–14**	**2015–18**	**All years**	Change (%)*
**MASI**							
Male		36·1%	36·7%	37·3%	38·1%	37·0%	5·5%
Female		31·6%	32·7%	34·2%	35·5%	33·4%	12·3%
**YLLI**							
Male		1·73	1·60	1·46	1·34	1·53	–22·5%
Female		0·97	0·91	0·86	0·82	0·89	–15·5%
**Standardised premature mortality rates per 100 000 person-years**							
Male							
	Most deprived†	865	796	725	694	770	–19·8%
	Least deprived‡	331	293	259	239	280	–27·8%
	Difference	534	503	465	455	489	–14·8%
Female							
	Most deprived†	511	478	449	443	470	–13·3%
	Least deprived‡	220	197	176	164	189	–25·5%
	Difference	291	281	272	279	281	–4·1%

Mortality is adjusted to the European Standard Population 2013. Rates for other deciles are not shown for brevity, and lie monotonically between these values. MASI=mortality attributable to socioeconomic inequality. YLLI=mean premature years of life lost per person.

* Change in values from 2003–06 to 2015–18.

† Most deprived refers to the most deprived decile of the index of multiple deprivation 2015.

‡ Least deprived refers to the least deprived decile.

If everyone in England had the same risk of mortality as the least deprived group, 877 082 fewer premature deaths would have happened between 2003 and 2018. This value equates to an average of one death every 10 min. 35·6% (95% CI 35·3–35·9) of premature deaths can be attributed to socioeconomic inequality.

**Figure 1:**
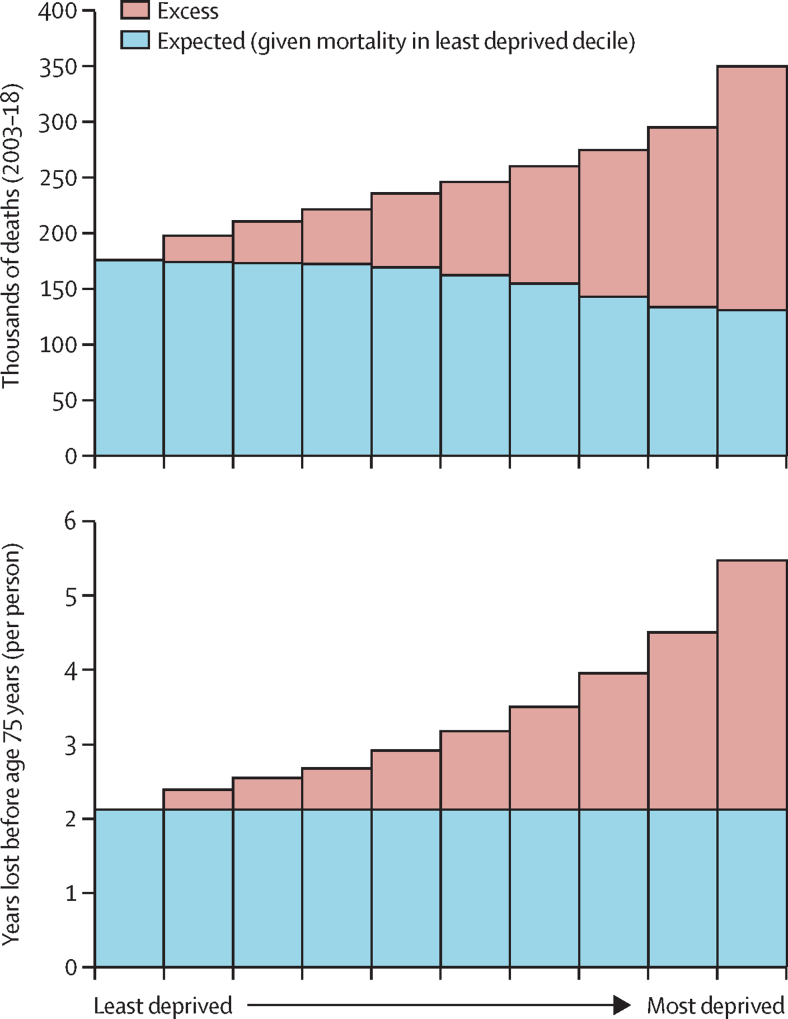
Mortality attributable to socioeconomic inequality and years lost to inequality in England, 2003–18 by index of multiple deprivation decile

The life table modelling suggested that premature mortality causes a mean of 3·3 years lost before age 75 years per person. If everyone had the mortality risk of the least deprived group, this value would be reduced to a mean of 2·1 years. Using this method, we estimate that 36·2% of years of life lost due to premature mortality can be attributed to inequality. Fewer deaths were expected in more deprived groups because they had a younger mean age (population pyramids in appendix p 2; figure 1).

**Figure 2:**
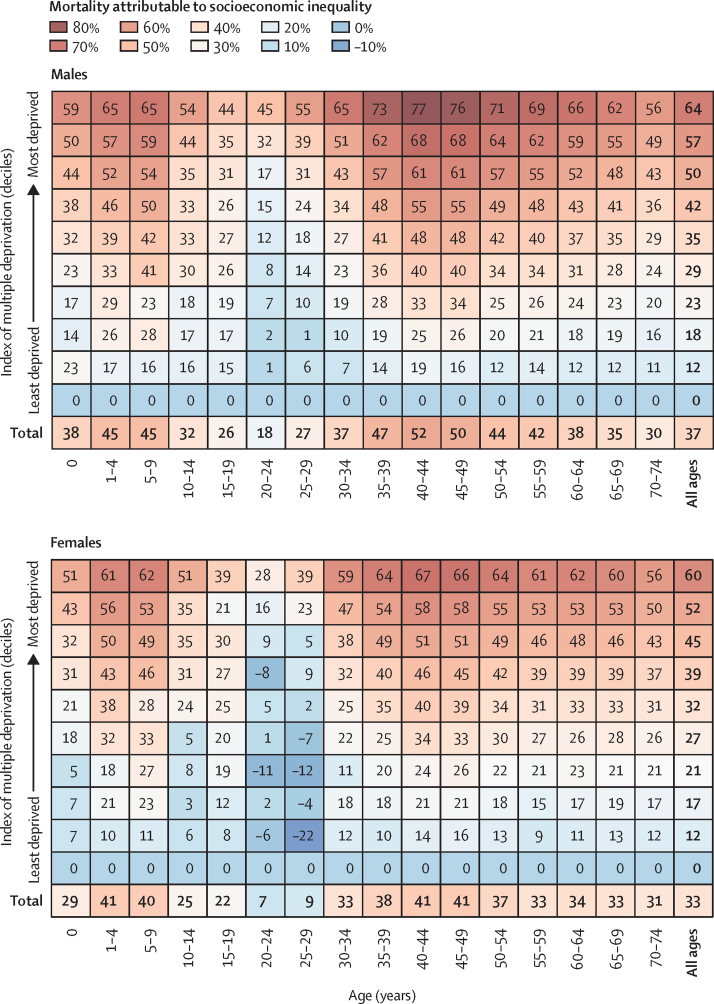
Percentage of premature mortality attributable to socioeconomic inequality by sex, deprivation, and age group in England, 2003–18 Values for the least deprived group are zero as this is the reference group.

The proportion of premature deaths attributable to inequality varies substantially by sex and age. The proportion of deaths attributable to socioeconomic inequality is higher in men (37%) than in women (33%) and peaks in early childhood (1–9 years) and middle age (40–49 years). In the most deprived areas, over three-quarters of deaths in men aged 35–49 years are attributable to socioeconomic inequalities (figure 2).

Of the 877 082 premature deaths attributable to socioeconomic inequality, the diseases that contributed most deaths were ischaemic heart disease (152 171, 17%), respiratory cancers (111 083, 13%), chronic obstructive pulmonary disease (COPD; 83 593, 10%), and digestive cancers (52 462, 6%; table 2).

**Table 2:** MASI and YLLI, by major cause of death in England (2003–18)

		**Premature deaths**				**Mean months of life lost per person before age 75 years**			
		Observed	Expected	Excess	MASI (95% CI)	Observed	Expected	Excess	YLLI (%)
Cancers									
	Breast	84 301	80 613	3 688	4·4% (2·5–6·2)	1·4	1·4	0·0	2·0%
	Digestive	271 981	219 519	52 462	19·3% (18·3–20·3)	3·4	2·8	0·6	17·9%
	Female genital	55 661	46 166	9495	17·1% (14·9–19·3)	0·8	0·7	0·1	18·5%
	Respiratory	236 968	125 885	111 083	46·9% (46·0–47·7)	2·7	1·4	1·3	48·1%
	Other cancers	73 354	67 936	5418	7·4% (5·4–9·4)	1·0	1·0	0·1	5·6%
	Lymphoid and haemopoietic	278 852	234 809	44 043	15·8% (14·8–16·8)	3·9	3·4	0·5	12·1%
Cardiovascular									
	Stroke	111 210	68 383	42 827	38·5% (37·2–39·8)	1·4	0·8	0·6	42·6%
	Ischaemic heart disease	335 526	183 355	152 171	45·4% (44·6–46·1)	4·1	2·1	2·0	48·4%
	Other heart disease	59 333	35 861	23 472	39·6% (37·7–41·4)	0·9	0·6	0·4	39·5%
	Other cardiovascular disease	97 475	59 376	38 099	39·1% (37·7–40·5)	1·3	0·7	0·6	43·9%
Nervous system		85 297	68 391	16 906	19·8% (18·0–21·5)	1·5	1·1	0·4	25·0%
Respiratory									
	COPD	129 062	45 469	83 593	64·8% (63·8–65·7)	1·3	0·4	0·9	68·5%
	Flu and pneumonia	57 676	28 163	29 513	51·2% (49·5–52·9)	0·8	0·4	0·4	53·5%
	Other respiratory causes	43 721	26 472	17 249	39·5% (37·3–41·6)	0·6	0·3	0·3	44·9%
Digestive									
	Liver	93 505	43 344	50 161	53·6% (52·4–54·9)	1·9	0·8	1·1	58·6%
	Other digestive disease	69 904	36 952	32 952	47·1% (45·6–48·7)	1·0	0·5	0·5	51·2%
External									
	Accidents	86 270	50 534	35 736	41·4% (39·8–43·1)	2·7	1·7	1·0	37·9%
	Other external causes	79 799	52 275	27 524	34·5% (32·7–36·3)	2·5	1·7	0·9	34·2%
Neonatal		31 247	19 490	11 757	37·6% (34·6–40·6)	2·5	1·7	0·8	33·1%
Other		184 143	95 211	88 932	48·3% (47·3–49·3)	4·0	2·1	2·0	48·3%
Total		2 465 285	1 588 203	877 082	35·6% (35·3–35·9)	39·8	25·4	14·4	36·2%

Data are n, % (95% CI), or %. MASI=mortality attributable to socioeconomic inequality. YLLI=years of life lost to inequality. COPD=chronic obstructive pulmonary disease.

**Figure 3:**
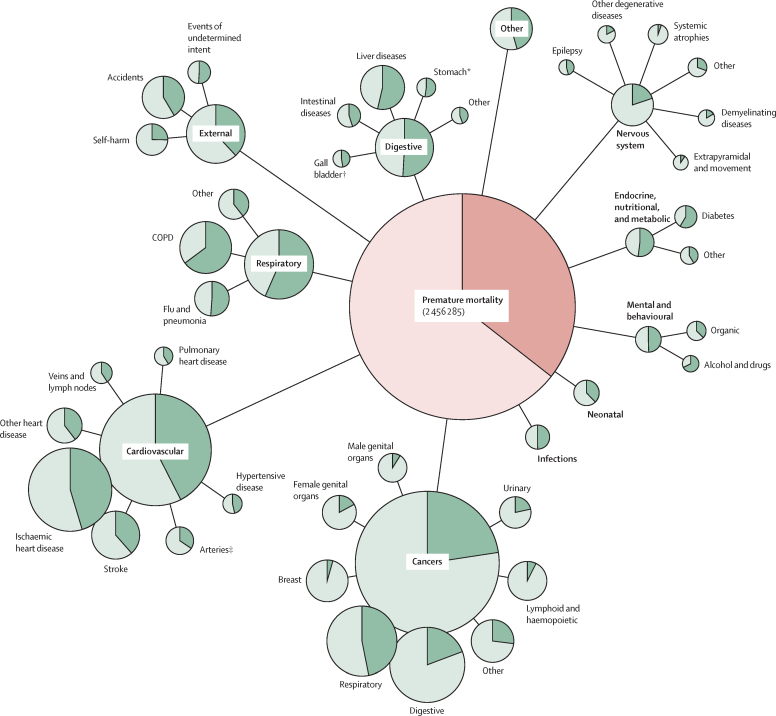
Inequality in premature mortality in England, 2003–18 Area of circles is proportional to the number of premature deaths. Dark areas represent deaths attributable to socioeconomic inequality. *Diseases of oesophagus, stomach, and duodenum. †Disorders of gallbladder, biliary tract, and pancreas. ‡Diseases of arteries, arterioles, and capillaries.

More than half of deaths for three major causes are attributable to inequality: COPD; liver disease; and flu and pneumonia (table 2, figure 3). Across all causes of death, the greatest inequality was observed for deaths due to tuberculosis, opioid use, HIV, psychoactive drug use, viral hepatitis, and obesity (table 2; appendix pp 3–7). Among cancers, which generally had low inequality, larynx, lung, and mouth cancers were exceptions, with high proportions of deaths attributable to inequality. The years of life lost expressed as a percentage was similar to MASI, across all diseases. Age of death did vary substantially by cause of death, and therefore specific diseases contributed more to the total years of life lost than to the total deaths attributable to inequality. These causes include alcohol and drugs, self harm, accidents, and particularly neonatal deaths, all of which typically occur at a younger age.

Sex-stratified results showed that inequality within major diseases was similar for men and women, with the greatest inequality for the same three diseases (COPD, liver disease, and flu and pneumonia; appendix p 8). The contribution of each major disease to the total number of attributable deaths was also similar for men and women, although ischaemic heart disease contributed a greater proportion for men (107 441 [20%] of 543 222 for men *vs* 44 729 [13%] of 333 860 for women).

At local authority level, the proportion of deaths attributable to socioeconomic inequality ranged from 58% in Manchester, an urban area in the north of England, to −13% (ie, 13% fewer deaths than the least deprived decile nationally experienced) in South Cambridgeshire, a rural area in the south of England. Areas of higher deprivation typically had higher values of MASI. An online interactive map of MASI is provided in appendix p 10, including inequality by cause of death for each local authority.

## Discussion

We did a cross-sectional study of 2·5 million premature deaths in England and found that one in three was attributable to neighbourhood deprivation measured by upstream determinants of health including income, employment, education, and crime. Inequality was greatest for respiratory, cardiovascular, and infectious diseases, whereas much less inequality was seen for cancers, except for cancers of the lung and mouth. Premature mortality reduced substantially during the study period but inequality persisted and increased among women because mortality due to non-cardiovascular causes plateaued or increased among the most deprived women.

Our study used data from the whole population of England to provide a population-level estimate of the scale of socioeconomic inequality in premature mortality. The method that we used to calculate MASI is replicable in other settings and can be used to make international comparisons and observe changes in inequality over time. Our data can also be broken down by disease category and geographical area to inform allocation of public health resources according to the absolute scale of inequality. By using data from the whole population, the method avoids selection bias.

The large proportion of premature deaths that could be avoided by eliminating socioeconomic differences in mortality is likely to exist across high-income countries, because inequalities in premature mortality are ubiquitous. Smaller cross-sectional studies (none in the UK, to our knowledge) have previously reported the proportion of premature deaths attributable to area-based deprivation and found similar values to our headline MASI value (36%), with values ranging from 22% to 42%.^5^, ^18^, ^19^, ^20^, ^21^, ^22^ The proportion is likely to be greater in countries with more extreme socioeconomic inequality, such as the USA, Spain, and Australia. Our cause-specific results might not be generalisable to other countries because the contribution of diseases to inequalities in mortality can differ substantially even where total inequalities are similar.^23^

We aimed to measure the extent of inequality in premature mortality rather than a causal effect of deprivation on mortality. The method is not designed to show that either inequality in isolation (which might affect health through the psychological stress of low socioeconomic position, for example) or any element of the IMD (such as the quality of local education and living environments) have caused premature deaths; or that improvements in the indicators that comprise the IMD would lead to fewer premature deaths. Instead, the study shows a scenario in which everyone in England has the same mortality of the least deprived decile, without showing how that scenario would be achieved. As such, the approach is designed to show the scale rather than the causes of inequality.

Nonetheless, the results of this study can help to understand some of the relations between deprivation and health. The IMD measures upstream determinants of health, and their effect on mortality is likely to be mediated by factors such as tobacco smoking, harmful alcohol consumption, hypertension, and obesity. We observed the greatest inequality for diseases with large behavioural components, such as lung cancer and liver disease, suggesting that health behaviours are important mediating pathways. Other studies have estimated the proportion of premature deaths that can be attributed to these intermediate factors. For example, a meta-analysis^11^ of cohort studies in seven high-income countries estimated that an average of 29% of premature deaths were attributable to smoking. People might smoke because they live in deprived circumstances and therefore some of these deaths could also be attributed to deprivation. These attributable fractions therefore overlap to some extent, and if fractions are calculated for several variables in the same causal pathway, the total could be more than 100%.

Our results also provide insight into changing inequality over time. Cardiovascular mortality reduced radically during the study period, which is part of a longer-term trend in the UK.^24^ Although all-cause premature mortality reduced across deprivation groups, higher socioeconomic groups had greater proportional reductions and therefore MASI increased (table 1). By contrast, absolute reductions in mortality were greatest for the most deprived groups, particularly for men. However, among the most deprived women, improvements in mortality were limited outside of cardiovascular diseases. For other diseases, such as COPD, mortality among the most deprived women actually increased. Premature mortality due to lung cancer among the most deprived men reduced between 2003–06 and 2015–18, while staying constant for the most deprived women (appendix pp 12, 13). This finding might relate to differences in the history of the male and female smoking epidemics, with smoking prevalence peaking earlier and reducing faster among men such that the prevalences converged between 1960 and 1990.^25^ Although we are not able to fully explain why time trends in inequality differed for men and women, the results show that plateauing or worsening premature mortality for non-cardiovascular diseases among the most deprived women underlies the large increase in MASI for women. Understanding and addressing this trend should be a public health priority.

Our analysis accounted for differences in age and sex between deprivation groups but we were unable to adjust for ethnicity because this characteristic is not recorded on death certificates in England. Ethnicity might be a confounder for diseases that have strong genetic or migration-related risk factors that are also associated with ethnicity. This confounder might be the case for tuberculosis, for example, where first generation migrants from countries with high incidence of tuberculosis are more likely to live in deprived areas. Analysis of census-linked data, such as the Census Longitudinal Study in the UK,^26^ could be used to assess the role of ethnicity in MASI.

Our analysis also does not account for selective internal migration. This factor might cause indicators to be understated if people move to wealthier areas when older or in poor health, or overstated if people move to poorer areas at these life stages, and could undermine sub-national results. Existing evidence suggests that people in poor health are more likely to move to deprived areas. Net internal immigration of people in poor health has been observed in particular in poor coastal towns in England.^27^ However, internal migration appears to play a minor role in social gradients in health.^28^, ^29^

The scale of premature mortality associated with neighbourhood deprivation shows the importance of addressing upstream determinants of health. Many mechanisms through which socioeconomic deprivation might harm health have been proposed, including inability to buy adequate food, housing, or health care; coping with behaviours such as alcohol consumption and smoking; differing cultural norms relating to healthy and unhealthy behaviours; stress and feelings of worthlessness associated with low socioeconomic position, which can lead to harmful physiological changes; lower social capital in deprived communities; environmental factors such as busy, polluting roads, fast food outlets, and waste disposal sites; poor prenatal and early childhood conditions causing poor health in adulthood; and social selection—a form of reverse causality in which sickness causes poverty.^30^ The relative importance of these mechanisms is debated and is beyond the scope of this study. The wide inequality in causes of death related to behavioural risk factors, and particularly alcohol, tobacco, and illicit drugs, highlights the importance of interventions that help people live healthier lifestyles. Evidence suggests that public health interventions such as smoking cessation that aim to change individual behaviour, although effective, can increase health inequalities as they are more likely to be adopted by people of higher socioeconomic position.^31^ Structural interventions such as taxation and minimum unit pricing are likely to have a more progressive effect.^32^

In the UK in 2018, disposable income for the richest quintile was 5·2 times the poorest quintile.^33^ Fiscal and welfare policies that enable or exacerbate this inequality are perhaps the highest priority targets to address the 877 082 excess deaths that we observed between 2003 and 2018. As well as addressing income inequality, the Marmot Review of health inequalities in 2010^34^ identified six policy objectives: giving every child the best start in life; enabling people to maximise their capabilities and have control; creating employment and good work; ensuring a healthy standard of living; creating healthy and sustainable places; and strengthening prevention of ill health. Our results show the high inequality in mortality among infants and young children and therefore the importance of objectives relating to early life.

Every year tens of thousands of premature deaths in England alone could be avoided by closing socioeconomic inequalities in mortality. Cardiovascular mortality has reduced but stalling of improvements for other disease categories, particularly for women living in deprived areas, needs further investigation. Local public health action and national policies need to prioritise interventions that address upstream determinants of these inequalities.

**This online publication has been corrected. The corrected version first appeared at thelancet.com/public-health on January 3, 2020**
