# Correction to *Lancet Public Health* 2020; 5: e33–41

**DOI:** 10.1016/S2468-2667(19)30256-7

**Published:** 2020-01-03

*Lewer D, Jayatunga W, Aldridge R, et al. Premature mortality attributable to socioeconomic inequality in England between 2003 and 2018: an observational study.* Lancet Public Health *2020; **5**: e33–41*—In the methods section of the summary in this Article, mortality rates has been changed to mortality. This correction has been made as of Jan 3, 2020.

