# Supplementary material for: Premature mortality attributable to socioeconomic inequality in England between 2003 and 2018: an observational study
Source: Lancet Public Health. 2019 Dec 5;5(1):e33–41. doi: 10.1016/S2468-2667(19)30219-1 (PMC7098478; doi:10.1016/S2468-2667(19)30219-1)
Supplement: Supplementary appendix [file mmc1.pdf]

THE LANCET
Public Health

Supplementary appendix

This appendix formed part of the original submission and has been peer reviewed. We post it as supplied by the authors.

Supplement to: Lewer D, Jayatunga W, Aldridge RW, et al. Premature mortality attributable to socioeconomic inequality in England between 2003 and 2018: an observational study. *Lancet Public Health 2019;* published online Dec 5. https://doi.org/10.1016/S2468-2667(19)30219-1.

Supplementary Information

1. Population pyramids by deprivation decile
2. Detailed cause-specific inequality
3. Mortality attributable to socioeconomic inequality, by major cause of death and sex
4. Map of mortality attributable to socioeconomic inequality, by local authority
5. Mortality attributable to socioeconomic inequality, using different quantiles of deprivation
6. Change in premature mortality rates between 2003-2006 and 2015-2018, by cause, sex and deprivation
7. Survival curves by sex and deprivation, estimated by life table modelling
8. References for supplementary information

1 Population pyramids by deprivation decile

Figure S1: Population pyramids by index of multiple deprivation 2015, aggregated for England, 2003-2018

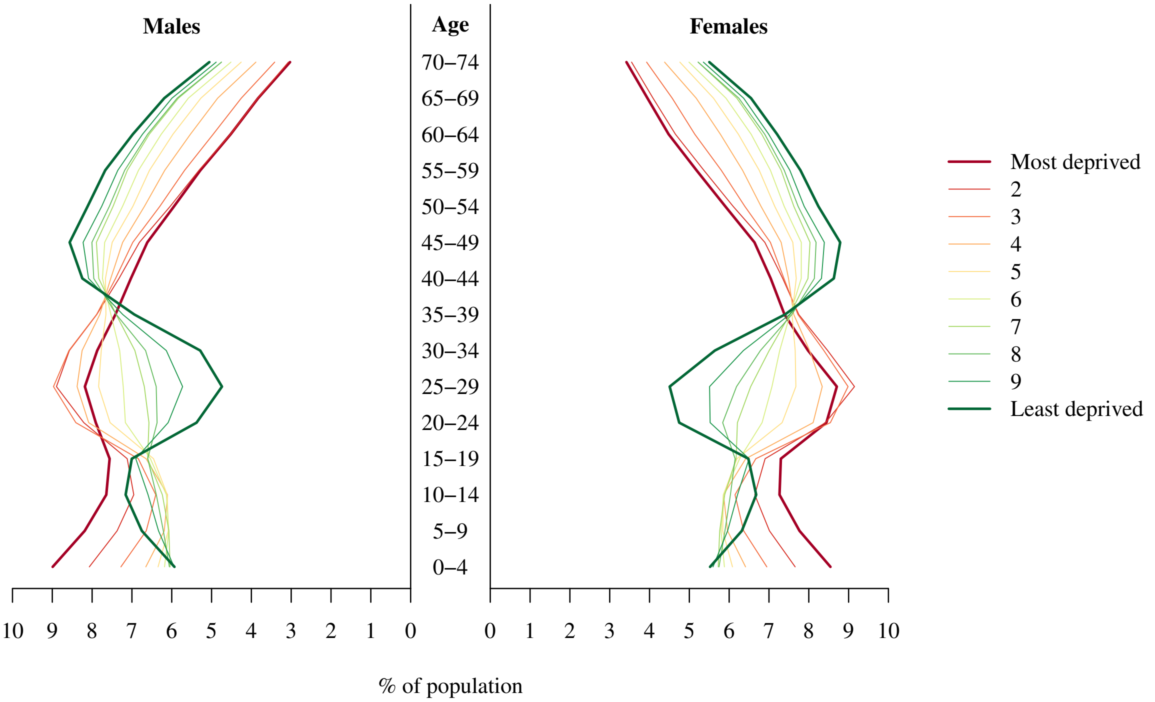

Populations are taken from publicly available mid-year population estimates produced by the Office for National Statistics [1]

2 Detailed cause-specific inequality (table S1)

Chapter	Subgroup	Diagnosis	ICD10 codes	Deaths	Expected deaths	Excess deaths	% of excess deaths	MASI (95% CI)	YLLs	Expected YLLs	Excess YLLs	% of exess YLLs	YLLI (%)
I Infections	Tuberculosis		A15-19	2,111	412	1,699	0.19%	80.5% (74.7 to 86.0)	6,966	1,008	5,959	0.25%	85.5%
	HIV		B20-24	2,907	707	2,200	0.25%	75.7% (69.8 to 81.2)	13,885	3,056	10,830	0.45%	78.0%
	Viral hepatitis		B15-19	2,717	854	1,863	0.21%	68.6% (62.2 to 74.8)	9,726	2,862	6,865	0.29%	70.6%
	Intestinal		A01-09	4,037	2,140	1,897	0.22%	47.0% (40.4 to 53.5)	8,289	4,326	3,963	0.17%	47.8%
	Other		A22-79; A96-98; B00-08; B25-99	14,793	8,512	6,282	0.72%	42.5% (38.8 to 46.0)	56,084	32,069	24,015	1.00%	42.8%
	Viral infections of the CNS		A81-89	1,551	1,450	101	0.01%	6.5% (-7.7 to 20.3)	4,732	4,433	299	0.01%	6.3%
	TOTAL		B00-99; A01-98	28,116	14,075	14,041	1.60%	49.9% (47.5 to 52.4)	99,683	47,753	51,930	2.16%	52.1%
II Cancers	Respiratory	Larynx	C32	5,957	2,347	3,610	0.41%	60.6% (56.0 to 65.2)	12,912	4,929	7,984	0.33%	61.8%
		Lung	C34	228,586	121,416	107,170	12.22%	46.9% (46.0 to 47.7)	437,024	226,221	210,803	8.78%	48.2%
		Other	C30-31; C33; C35-39	2,425	2,121	304	0.03%	12.5% (1.8 to 22.9)	6,688	5,959	728	0.03%	10.9%
		Total	C30-39	236,968	125,885	111,083	12.67%	46.9% (46.0 to 47.7)	456,624	237,109	219,515	9.14%	48.1%
	Mouth		C00-14	20,322	10,987	9,335	1.06%	45.9% (43.0 to 48.8)	52,289	27,763	24,527	1.02%	46.9%
	Other		C76-80; C97; D00-48	80,830	57,854	22,976	2.62%	28.4% (26.8 to 30.1)	175,316	129,855	45,461	1.89%	25.9%
	Urinary	Bladder	C67	20,910	15,085	5,825	0.66%	27.9% (24.6 to 31.2)	35,838	25,719	10,119	0.42%	28.2%
		Other	C65-66; C68	1,753	1,374	379	0.04%	21.6% (10.0 to 33.2)	3,070	2,318	752	0.03%	24.5%
		Kidney	C64	25,151	21,026	4,125	0.47%	16.4% (13.1 to 19.6)	56,494	47,697	8,797	0.37%	15.6%
		Total	C64-68	47,814	37,485	10,329	1.18%	21.6% (19.3 to 23.8)	95,402	75,734	19,668	0.82%	20.6%
	Digestive	Anus and anal canal	C21	2,281	1,398	883	0.10%	38.7% (29.3 to 47.8)	5,907	3,615	2,292	0.10%	38.8%
		Stomach	C16	26,583	17,911	8,672	0.99%	32.6% (29.7 to 35.4)	55,608	38,887	16,720	0.70%	30.1%
		Gallbladder	C23	2,830	1,981	849	0.10%	30.0% (21.2 to 38.8)	5,523	3,888	1,635	0.07%	29.6%
		Liver & IH bile ducts	C22	27,129	19,451	7,678	0.88%	28.3% (25.4 to 31.1)	57,915	39,461	18,454	0.77%	31.9%
		Oesophagus	C15	51,072	37,149	13,924	1.59%	27.3% (25.2 to 29.4)	105,586	77,115	28,471	1.19%	27.0%
		Rectum	C20	23,833	19,337	4,496	0.51%	18.9% (15.6 to 22.1)	50,310	42,573	7,737	0.32%	15.4%
		Other	C26	14,741	12,551	2,190	0.25%	14.9% (10.6 to 19.1)	30,360	26,798	3,562	0.15%	11.7%
		Pancreas	C25	54,664	47,392	7,272	0.83%	13.3% (11.1 to 15.5)	107,582	93,865	13,718	0.57%	12.8%
		Rectosigmoid junction	C19	13,362	11,778	1,584	0.18%	11.9% (7.2 to 16.4)	29,257	27,514	1,743	0.07%	6.0%
		Biliary tract	C24	1,348	1,210	138	0.02%	10.2% (-3.9 to 24.3)	2,584	2,028	557	0.02%	21.5%
		Small intestine	C17	2,780	2,523	257	0.03%	9.3% (-0.9 to 19.3)	6,093	5,445	648	0.03%	10.6%
		Colon	C18	51,358	46,837	4,521	0.52%	8.8% (6.5 to 11.1)	104,154	99,326	4,829	0.20%	4.6%
		Total	C15-26	271,981	219,519	52,462	5.98%	19.3% (18.3 to 20.3)	560,880	460,514	100,365	4.18%	17.9%
	Female genital organs		C51-58	55,661	46,166	9,495	1.08%	17.1% (14.9 to 19.2)	133,014	108,386	24,628	1.03%	18.5%
	Thyroid & endocrine glands		C73-75	3,906	3,438	468	0.05%	12.0% (3.4 to 20.5)	14,862	13,128	1,734	0.07%	11.7%
	Male genital organs		C60-63	39,842	36,270	3,572	0.41%	9.0% (6.3 to 11.5)	58,340	54,937	3,403	0.14%	5.8%
	Lymphoid & haematopoietic	Leukaemia, unspecified	C95	1,089	901	188	0.02%	17.3% (1.0 to 32.9)	3,128	3,059	69	0.00%	2.2%
		Hodgkin lymphoma	C81	2,543	2,109	434	0.05%	17.1% (5.9 to 27.9)	9,360	8,853	506	0.02%	5.4%
		Follicular lymphoma	C82	1,298	1,155	143	0.02%	11.0% (-3.3 to 25.1)	2,770	2,534	236	0.01%	8.5%

Chapter	Subgroup	Diagnosis	ICD10 codes	Deaths	Expected deaths	Excess deaths	% of excess deaths	MASI (95% CI)	YLLs	Expected YLLs	Excess YLLs	% of exess YLLs	YLLI (%)
		Other	C85-89; C93-94; C96	19,650	17,581	2,069	0.24%	10.5% (6.7 to 14.3)	42,563	38,342	4,221	0.18%	9.9%
		T/NK-cell lymphomas	C84	2,341	2,114	227	0.03%	9.7% (-1.6 to 20.4)	6,394	5,275	1,119	0.05%	17.5%
		Myeloid leukaemia	C92	17,733	16,316	1,418	0.16%	8.0% (3.9 to 12.0)	45,224	43,166	2,057	0.09%	4.5%
		Lymphoid leukaemia	C91	7,387	7,014	373	0.04%	5.0% (-1.4 to 11.4)	23,781	22,870	911	0.04%	3.8%
		Multiple myeloma	C90	15,428	14,880	548	0.06%	3.6% (-0.8 to 7.9)	27,827	26,529	1,298	0.05%	4.7%
		Non-follicular lymphoma	C83	5,885	5,866	19	0.00%	0.3% (-6.8 to 7.3)	13,840	14,378	-538	-0.02%	-3.9%
		Total	C81-96	73,354	67,936	5,418	0.62%	7.4% (5.3 to 9.4)	174,888	165,009	9,879	0.41%	5.6%
	Bone and articular cartilage		C40-41	2,880	2,703	177	0.02%	6.1% (-5.3 to 16.9)	16,126	16,886	-760	-0.03%	-4.7%
	Mesothelial and soft tissue		C45-49	25,384	24,246	1,138	0.13%	4.5% (1.1 to 7.9)	55,476	54,844	632	0.03%	1.1%
	Breast		C50	84,301	80,613	3,688	0.42%	4.4% (2.5 to 6.2)	230,984	226,387	4,597	0.19%	2.0%
	Eye, brain & CNS		C69-72	38,626	40,965	-2,339	-0.27%	-6.1% (-9.1 to -3.1)	124,255	134,618	-10,364	-0.43%	-8.3%
	Skin		C43-44	19,248	20,862	-1,614	-0.18%	-8.4% (-12.6 to -4.1)	51,695	58,265	-6,570	-0.27%	-12.7%
	TOTAL		C00-97; D00-48	1,001,117	774,927	226,190	25.79%	22.6% (22.1 to 23.1)	2,200,151	1,763,434	436,717	18.19%	19.8%
III Blood	Aplastic & other anaemias		D60-64	972	576	396	0.05%	40.7% (26.2 to 54.4)	3,077	1,838	1,239	0.05%	40.3%
	Immune mechanism		D80-89	2,032	1,251	781	0.09%	38.4% (28.1 to 48.3)	7,025	4,640	2,386	0.10%	34.0%
	Other		D50-59; D65-77	3,348	2,323	1,025	0.12%	30.6% (22.2 to 39.2)	13,290	8,409	4,881	0.20%	36.7%
	TOTAL		D50-89	6,352	4,151	2,202	0.25%	34.7% (28.8 to 40.6)	23,392	14,887	8,506	0.35%	36.4%
IIV Endocrine, nutritional & metabolic	Obesity		E66-67	4,393	1,414	2,979	0.34%	67.8% (62.7 to 72.7)	14,936	4,242	10,694	0.45%	71.6%
	Diabetes mellitus		E10-14	23,787	9,848	13,939	1.59%	58.6% (56.1 to 61.0)	57,606	22,786	34,820	1.45%	60.4%
	Other		E00-07; E15-63	1,931	1,013	918	0.10%	47.5% (37.8 to 57.1)	6,569	3,157	3,412	0.14%	51.9%
	Metabolic -disorders	Other	E70-83; E86-88	5,027	2,836	2,191	0.25%	43.6% (37.1 to 49.9)	28,757	18,160	10,598	0.44%	36.9%
		Amyloidosis	E85	1,969	1,865	104	0.01%	5.3% (-6.9 to 16.6)	3,650	3,335	316	0.01%	8.6%
		Cystic fibrosis	E84	1,556	1,592	-36	0.00%	-2.3% (-19.9 to 14.6)	12,404	12,698	-294	-0.01%	-2.4%
		Total	E70-88	8,552	6,292	2,260	0.26%	26.4% (20.7 to 32.0)	44,812	34,193	10,619	0.44%	23.7%
	TOTAL		E00-88	38,663	18,567	20,096	2.29%	52.0% (49.9 to 54.1)	123,922	64,378	59,545	2.48%	48.0%
V Mental & behavioural	Psychoactive substance use	Opioids	F11	3,165	696	2,469	0.28%	78.0% (72.0 to 83.7)	20,202	5,347	14,856	0.62%	73.5%
		Other	F12-19	2,710	687	2,023	0.23%	74.6% (67.7 to 81.3)	17,252	5,303	11,949	0.50%	69.3%
		Alcohol	F10	7,190	2,846	4,344	0.50%	60.4% (56.0 to 64.7)	27,516	10,825	16,690	0.70%	60.7%
		Total	F10-19	13,065	4,229	8,837	1.01%	67.6% (64.5 to 70.8)	64,969	21,475	43,495	1.81%	66.9%
	Organic		F01-09	17,788	11,133	6,655	0.76%	37.4% (34.1 to 40.6)	17,559	11,582	5,977	0.25%	34.0%
	Other		F20-99	1,100	750	350	0.04%	31.8% (16.3 to 46.6)	4,717	3,311	1,406	0.06%	29.8%
	TOTAL		F01-99	31,953	16,112	15,841	1.81%	49.6% (47.3 to 51.8)	87,245	36,367	50,878	2.12%	58.3%
VI Nervous	Episodic & paroxysmal		G40-47	11,002	5,997	5,005	0.57%	45.5% (41.0 to 49.9)	57,627	34,485	23,142	0.96%	40.2%
	Myoneural junction & muscle		G70-72	3,689	2,436	1,253	0.14%	34.0% (25.9 to 42.0)	19,148	12,173	6,975	0.29%	36.4%
	Other		G00-09; G50-64; G90-98	9,050	6,289	2,761	0.31%	30.5% (25.4 to 35.5)	34,939	23,467	11,472	0.48%	32.8%
	Cerebral palsy & paralytic		G80-83	4,256	3,068	1,188	0.14%	27.9% (19.8 to 35.9)	26,398	20,746	5,652	0.24%	21.4%
	Other degenerative		G30-31	14,755	12,202	2,553	0.29%	17.3% (13.1 to 21.4)	22,703	18,304	4,399	0.18%	19.4%

Chapter	Subgroup	Diagnosis	ICD10 codes	Deaths	Expected deaths	Excess deaths	% of excess deaths	MASI (95% CI)	YLLs	Expected YLLs	Excess YLLs	% of exess YLLs	YLLI (%)
	Demyelinating diseases of CNS		G35-37	12,872	10,738	2,134	0.24%	16.6% (12.1 to 21.0)	35,443	29,322	6,121	0.25%	17.3%
	Extrapyramidal & movement		G20-26	10,665	9,671	994	0.11%	9.3% (4.2 to 14.3)	11,281	9,837	1,444	0.06%	12.8%
	Systemic atrophies of CNS		G10-14	19,008	17,992	1,016	0.12%	5.3% (1.5 to 9.2)	47,214	42,751	4,463	0.19%	9.5%
	TOTAL		G00-98	85,297	68,391	16,906	1.93%	19.8% (18.1 to 21.5)	254,753	191,085	63,669	2.65%	25.0%
IX Circulatory	Hypertensive	Renal	I12	1,286	672	614	0.07%	47.8% (36.0 to 58.8)	2,278	1,049	1,229	0.05%	53.9%
		Other	I10; I13-15	2,208	1,163	1,045	0.12%	47.3% (38.6 to 56.0)	4,251	2,162	2,089	0.09%	49.1%
		Heart failure	I11	14,467	7,809	6,658	0.76%	46.0% (42.5 to 49.4)	32,338	15,510	16,828	0.70%	52.0%
		Total	I10-15	17,961	9,644	8,318	0.95%	46.3% (43.2 to 49.4)	38,867	18,721	20,146	0.84%	51.8%
	Ischaemic heart diseases	Other	I20; I22-24	1,320	618	702	0.08%	53.2% (42.4 to 63.8)	2,691	1,110	1,581	0.07%	58.8%
		Chronic	I25	194,952	103,665	91,288	10.41%	46.8% (45.9 to 47.7)	393,748	196,253	197,495	8.22%	50.2%
		Acute MI	I21	139,254	79,072	60,182	6.86%	43.2% (42.1 to 44.4)	290,400	157,190	133,210	5.55%	45.9%
		Total	I20-25	335,526	183,355	152,171	17.35%	45.4% (44.6 to 46.1)	686,839	354,553	332,286	13.84%	48.4%
	Pulmonary	Other	I27-28	2,960	1,690	1,270	0.14%	42.9% (34.7 to 50.9)	9,202	5,801	3,401	0.14%	37.0%
		Pulmonary embolism	I26	13,314	7,882	5,432	0.62%	40.8% (37.0 to 44.6)	31,475	17,626	13,849	0.58%	44.0%
		Total	I26-28	16,274	9,573	6,701	0.76%	41.2% (37.7 to 44.6)	40,677	23,427	17,250	0.72%	42.4%
	Veins, lymph vessels & nodes	Other	I81-89	2,739	1,264	1,475	0.17%	53.9% (46.3 to 61.3)	9,035	3,358	5,677	0.24%	62.8%
		Phlebitis & thrombo.	I80	19,324	11,755	7,569	0.86%	39.2% (35.9 to 42.3)	48,857	26,012	22,845	0.95%	46.8%
		Total	I80-89	22,063	13,019	9,044	1.03%	41.0% (38.0 to 43.9)	57,892	29,371	28,522	1.19%	49.3%
	Other types of -heart disease	Heart failure	I50	10,577	5,329	5,248	0.60%	49.6% (45.8 to 53.4)	16,971	7,677	9,294	0.39%	54.8%
		Cardiomyopathy	I42	14,646	9,085	5,561	0.63%	38.0% (34.2 to 41.7)	53,071	32,400	20,672	0.86%	39.0%
		Other	I30-40; I44-47; I49; I51	29,583	18,506	11,077	1.26%	37.4% (34.7 to 40.1)	82,759	52,107	30,652	1.28%	37.0%
		Atrial fibrillation & flutter	I48	4,527	2,941	1,586	0.18%	35.0% (28.2 to 41.7)	5,512	3,518	1,994	0.08%	36.2%
		Total	I30-51	59,333	35,861	23,472	2.68%	39.6% (37.7 to 41.4)	158,313	95,702	62,611	2.61%	39.5%
	Stroke	Other	I64-69	49,919	29,527	20,392	2.32%	40.8% (38.9 to 42.7)	73,290	39,621	33,669	1.40%	45.9%
		Infarction	I63	12,750	7,550	5,200	0.59%	40.8% (36.9 to 44.6)	26,021	15,049	10,971	0.46%	42.2%
		Haemorrhage	I60-62	48,541	31,306	17,235	1.97%	35.5% (33.4 to 37.6)	128,942	76,383	52,559	2.19%	40.8%
		Total	I60-69	111,210	68,383	42,827	4.88%	38.5% (37.2 to 39.8)	228,253	131,053	97,199	4.05%	42.6%
	Arteries	Other	I70; I72-78	9,477	4,548	4,929	0.56%	52.0% (47.9 to 55.9)	17,561	8,313	9,249	0.39%	52.7%
		Aortic aneurysm & dis.	I71	27,370	19,628	7,743	0.88%	28.3% (25.4 to 31.1)	45,148	32,420	12,729	0.53%	28.2%
		Total	I70-78	36,847	24,175	12,672	1.44%	34.4% (32.0 to 36.7)	62,710	40,732	21,977	0.92%	35.0%
	Other		I00-09; I95-99	4,330	2,966	1,364	0.16%	31.5% (24.2 to 38.6)	8,938	4,944	3,994	0.17%	44.7%
	TOTAL		I00-99	603,544	346,975	256,569	29.25%	42.5% (42.0 to 43.0)	1,282,489	698,504	583,985	24.32%	45.5%
X Respiratory	Chronic lung diseases	Other (COPD)	J40-44; J46-47	123,758	43,088	80,670	9.20%	65.2% (64.2 to 66.2)	201,237	59,618	141,618	5.90%	70.4%
		Asthma	J45	5,304	2,381	2,923	0.33%	55.1% (49.7 to 60.6)	20,054	10,068	9,987	0.42%	49.8%
		Total	J40-47	129,062	45,469	83,593	9.53%	64.8% (63.8 to 65.7)	221,291	69,686	151,605	6.31%	68.5%
	Lung abscess, gangrene, pyothorax		J85-86	2,103	877	1,226	0.14%	58.3% (50.1 to 66.2)	6,178	2,468	3,711	0.15%	60.1%
	Influenza & pneumonia		J09-18	57,676	28,163	29,513	3.36%	51.2% (49.5 to 52.8)	136,170	63,351	72,819	3.03%	53.5%
	Other lung infections		J20-22	4,351	2,245	2,106	0.24%	48.4% (41.9 to 54.6)	12,493	6,409	6,084	0.25%	48.7%

Chapter	Subgroup	Diagnosis	ICD10 codes	Deaths	Expected deaths	Excess deaths	% of excess deaths	MASI (95% CI)	YLLs	Expected YLLs	Excess YLLs	% of exess YLLs	YLLI (%)
	External agents		J60-70	7,912	4,255	3,658	0.42%	46.2% (41.4 to 50.8)	17,492	9,503	7,989	0.33%	45.7%
	Other		J00-06; J32-39; J80-84; J90-98	29,355	19,096	10,259	1.17%	34.9% (32.3 to 37.6)	57,451	33,163	24,289	1.01%	42.3%
	TOTAL		J00-98	230,459	100,104	130,355	14.86%	56.6% (55.8 to 57.3)	451,075	184,580	266,496	11.10%	59.1%
XI Digestive	Liver	Alcoholic liver disease	K70	63,105	27,650	35,455	4.04%	56.2% (54.7 to 57.7)	230,161	92,947	137,214	5.71%	59.6%
		Other	K71-73; K75-76	12,794	6,417	6,377	0.73%	49.8% (46.2 to 53.4)	40,081	16,679	23,402	0.97%	58.4%
		Fibrosis & cirrhosis	K74	17,606	9,277	8,330	0.95%	47.3% (44.0 to 50.4)	50,101	23,014	27,087	1.13%	54.1%
		Total	K70-76	93,505	43,344	50,161	5.72%	53.6% (52.4 to 54.9)	320,343	132,639	187,703	7.82%	58.6%
	Oesophagus, stomach, duodenum		K20-31	17,111	8,177	8,934	1.02%	52.2% (49.1 to 55.2)	42,779	18,820	23,959	1.00%	56.0%
	Hernia		K40-46	3,153	1,598	1,555	0.18%	49.3% (42.0 to 56.4)	6,469	3,209	3,260	0.14%	50.4%
	Gallbladder, biliary tract, pancreas		K80-87	13,877	7,314	6,564	0.75%	47.3% (43.8 to 50.8)	34,961	16,610	18,351	0.76%	52.5%
	Other		K00-14; K35-38; K65-66; K90-92	6,841	3,608	3,233	0.37%	47.3% (42.2 to 52.2)	16,135	8,090	8,046	0.34%	49.9%
	Other intestines		K55-64	25,674	14,116	11,558	1.32%	45.0% (42.3 to 47.6)	52,243	26,901	25,341	1.06%	48.5%
	Noninfective enteritis & colitis		K50-52	3,248	2,139	1,109	0.13%	34.1% (25.4 to 42.1)	8,532	5,038	3,493	0.15%	40.9%
	TOTAL		K00-92	163,409	80,296	83,113	9.48%	50.9% (49.9 to 51.9)	481,461	211,308	270,153	11.25%	56.1%
XII Skin & subcutaneous tissue	Other		L10-98	2,264	942	1,322	0.15%	58.4% (50.4 to 66.2)	4,800	2,398	2,402	0.10%	50.0%
	Infections		L00-08	3,711	1,909	1,803	0.21%	48.6% (41.6 to 55.1)	7,948	3,694	4,254	0.18%	53.5%
	TOTAL		L00-98	5,975	2,851	3,124	0.36%	52.3% (47.1 to 57.5)	12,747	6,092	6,656	0.28%	52.2%
XIII Musculoskeletal system & connective tissue	Osteopathies & chondropathies		M80-94	1,991	1,162	829	0.09%	41.6% (31.9 to 51.0)	3,422	1,727	1,694	0.07%	49.5%
	Soft tissue disorders		M60-79	1,690	995	695	0.08%	41.1% (30.2 to 51.4)	4,751	2,877	1,874	0.08%	39.4%
	Arthropathies		M00-25	5,239	3,334	1,905	0.22%	36.4% (30.0 to 42.5)	8,822	5,106	3,716	0.15%	42.1%
	Other		M40-54; M95-98	1,523	1,062	462	0.05%	30.3% (17.9 to 42.4)	4,997	3,234	1,763	0.07%	35.3%
	Systemic connective tissue		M30-35	3,970	2,851	1,119	0.13%	28.2% (20.3 to 35.9)	11,576	7,117	4,459	0.19%	38.5%
	TOTAL		M00-98	14,413	9,403	5,010	0.57%	34.8% (30.9 to 38.5)	33,568	20,062	13,506	0.56%	40.2%
XIV Genitourinary system	Renal tubulo-interstitial		N10-15	2,595	1,290	1,305	0.15%	50.3% (42.3 to 58.2)	6,504	3,387	3,117	0.13%	47.9%
	Other		N20-99	12,200	6,444	5,756	0.66%	47.2% (43.3 to 51.0)	22,293	11,086	11,207	0.47%	50.3%
	Renal failure		N17-19	6,240	3,303	2,937	0.33%	47.1% (41.8 to 52.3)	12,823	6,568	6,255	0.26%	48.8%
	Glomerular		N00-07	1,096	656	440	0.05%	40.2% (26.2 to 53.2)	2,864	2,214	650	0.03%	22.7%
	TOTAL		N00-99	22,131	11,693	10,438	1.19%	47.2% (44.3 to 50.0)	44,483	23,254	21,229	0.88%	47.7%
XVII Congenital	Nervous system		Q00-07	1,634	791	843	0.10%	51.6% (40.4 to 62.4)	14,329	7,120	7,209	0.30%	50.3%
	Chromosomal, NEC		Q90-99	4,575	2,479	2,096	0.24%	45.8% (39.2 to 52.1)	22,022	14,862	7,160	0.30%	32.5%
	Other		Q18; Q30-89	3,709	2,386	1,323	0.15%	35.7% (27.4 to 43.6)	27,109	16,643	10,466	0.44%	38.6%
	Circulatory system		Q20-28	5,608	4,100	1,508	0.17%	26.9% (19.8 to 34.0)	43,271	32,110	11,161	0.46%	25.8%
	TOTAL		Q00-99	15,526	9,756	5,770	0.66%	37.2% (33.1 to 41.0)	106,732	70,735	35,996	1.50%	33.7%
XVIII Symptoms, signs & abnormal findings			R02-99	17,305	6,473	10,832	1.24%	62.6% (59.7 to 65.4)	99,264	39,477	59,787	2.49%	60.2%
XX External	Undetermined intent		Y10-34; U50	24,739	12,154	12,585	1.43%	50.9% (48.1 to 53.7)	146,773	74,664	72,109	3.00%	49.1%

Chapter	Subgroup	Diagnosis	ICD10 codes	Deaths	Expected deaths	Excess deaths	% of excess deaths	MASI (95% CI)	YLLs	Expected YLLs	Excess YLLs	% of exess YLLs	YLLI (%)
	Accidents	Drugs & alcohol poisoning	X41-42; X44-45	25,928	9,066	16,862	1.92%	65.0% (62.6 to 67.5)	144,568	54,816	89,752	3.74%	62.1%
		Other	X00-40; X43; X46-59; W00-98; V00; V07-08; V37; V45; V57; V68; V80; V83; V86; V90-97	36,360	21,723	14,637	1.67%	40.3% (37.8 to 42.7)	145,441	86,483	58,959	2.46%	40.5%
		Vehicle accident	V01-06; V09-34; V38-44; V46-55; V58-67; V69-79; V81-82; V84-85; V87-89	23,982	19,745	4,237	0.48%	17.7% (13.9 to 21.4)	160,495	138,405	22,090	0.92%	13.8%
		Total	X00-59; V00-97; W00-98	86,270	50,534	35,736	4.07%	41.4% (39.8 to 43.1)	450,504	279,704	170,800	7.11%	37.9%
	Medical & surgical care		Y40-84	4,829	2,881	1,948	0.22%	40.3% (33.8 to 46.6)	14,101	8,151	5,950	0.25%	42.2%
	Other		X85-99; Y00-09; Y35-36; Y85-98	2,300	1,419	881	0.10%	38.3% (27.7 to 48.5)	12,325	8,045	4,281	0.18%	34.7%
	Intentional self-harm		X60-84	47,931	35,820	12,111	1.38%	25.3% (22.8 to 27.7)	248,961	187,001	61,961	2.58%	24.9%
	TOTAL		X00-99; V00-97; W00-98; Y00-98; U50	166,069	102,809	63,260	7.21%	38.1% (36.9 to 39.3)	872,665	557,564	315,100	13.12%	36.1%
Neonatal			All causes age 0-27 days	31,247	19,490	11,757	1.34%	37.6% (34.6 to 40.6)	422,093	282,303	139,790	5.82%	33.1%
Other			P00-96; O00-99; H05-82; Z05-ZZ; S18; T32-98; U00-49; U83-85	3,709	2,132	1,577	0.18%	42.5% (34.3 to 50.3)	42,970	25,624	17,346	0.72%	40.4%
ALL-CAUSE				2,465,285	1,588,203	877,082	100.00%	35.6% (35.3, 35.9)	6,638,694	4,237,406	2,401,288	100.00%	36.2%

3 Mortality attributable to socioeconomic inequality by major cause of death and sex

Figure S2: Mortality attributable to socioeconomic inequality by major cause of death and sex, 2003-2018

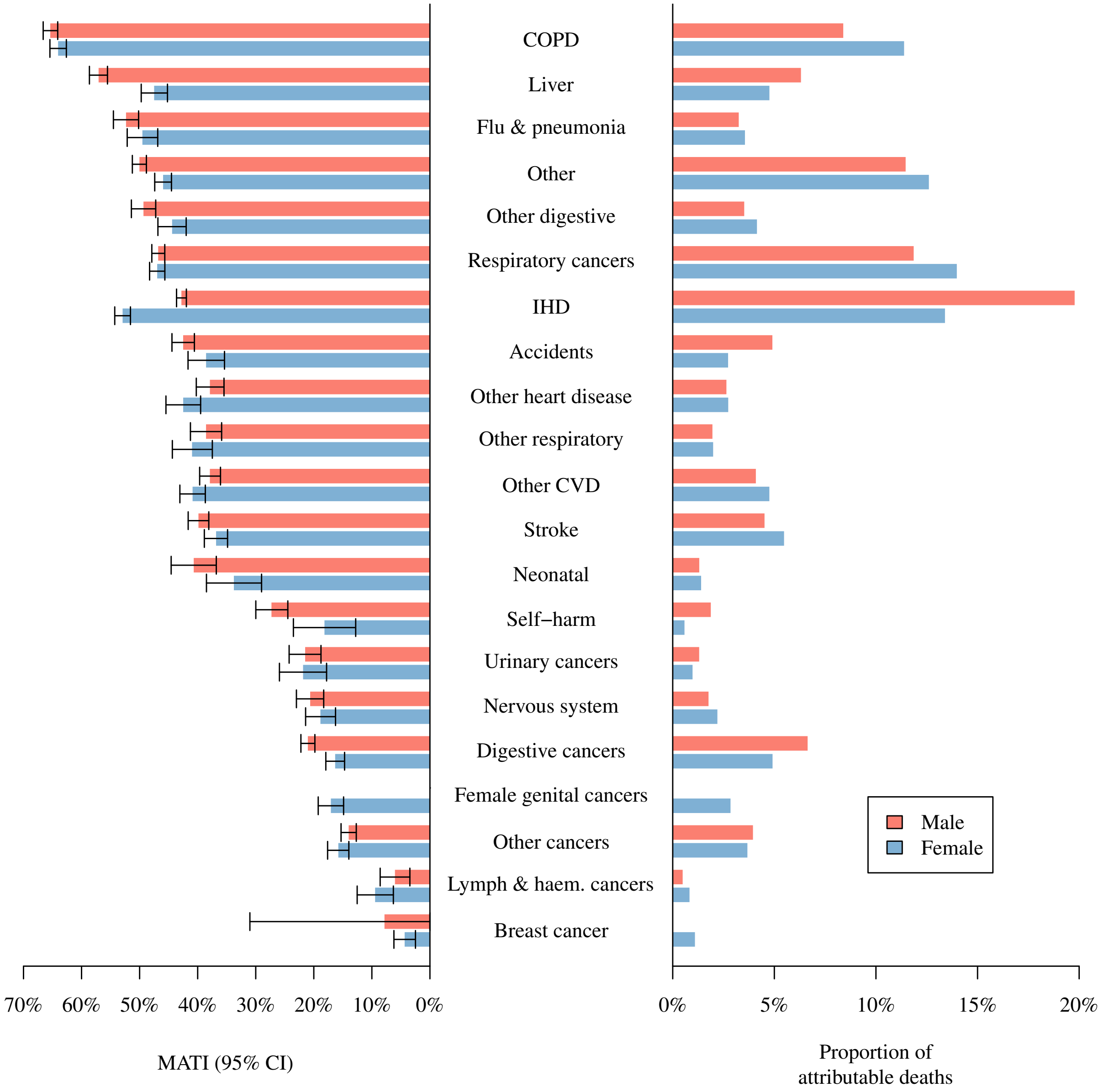

Table S2: Mortality attributable to socioeconomic inequality by major cause of death, 2003-2018: Males

			Deaths before age 75				Months of life lost before age 75			
Chapter	Major cause of death	ICD10 codes	Observed	Expected	Excess	MASI (95% CI)	Observed	Expected	Excess	YLLI (%)
Cancers	Breast	C50	501	462	39	7.8% (-17.0%, 30.7%)	<0.1	<0.1	<0.1	-4.90%
	Digestive	C15-26	171,504	135,444	36,060	21.0% (19.8%, 22.2%)	4.2	3.4	0.8	19.40%
	Female genital organs	C51-58	-	-	-	-	-	-	-	-
	Respiratory	C30-39	137,610	73,200	64,410	46.8% (45.7%, 47.9%)	3.1	1.6	1.5	47.70%
	Lymphoid & haematopoietic	C81-96	44,141	41,482	2,659	6.0% (3.5%, 8.5%)	1.3	1.2	0.1	2.10%
	Other		186,176	157,658	28,518	15.3% (14.1%, 16.5%)	4.9	4.3	0.6	11.80%
Circulatory	Stroke	I60-69	61,553	37,014	24,539	39.9% (38.1%, 41.6%)	1.5	0.9	0.7	43.10%
	IHD	I20-25	251,020	143,578	107,442	42.8% (42.0%, 43.7%)	6.4	3.5	2.9	45.40%
	Other		58,642	36,417	22,225	37.9% (36.0%, 39.7%)	1.5	0.9	0.6	42.50%
	Other heart diseases	I30-51	37,871	23,519	14,352	37.9% (35.5%, 40.2%)	1.3	0.8	0.5	37.70%
Neonatal			17,434	10,341	7,093	40.7% (36.7%, 44.6%)	2.8	1.8	1.0	36.40%
Other			102,480	51,269	51,211	50.0% (48.7%, 51.3%)	4.6	2.3	2.3	50.60%
Nervous system		G00-98	46,401	36,831	9,570	20.6% (18.3%, 22.9%)	1.7	1.3	0.4	26.20%
Respiratory	COPD	J40-44	69,711	24,128	45,583	65.4% (64.1%, 66.7%)	1.4	0.5	1.0	68.30%
	Flu & pneumonia	J09-18	33,716	16,069	17,647	52.3% (50.2%, 54.5%)	1.0	0.4	0.5	55.30%
	Other		27,518	16,907	10,611	38.6% (35.8%, 41.2%)	0.7	0.4	0.3	44.60%
Digestive	Liver	K70-76	60,067	25,789	34,278	57.1% (55.5%, 58.6%)	2.5	1.0	1.5	61.00%
	Other		38,747	19,631	19,116	49.3% (47.3%, 51.5%)	1.1	0.5	0.6	54.40%
External	Accidents	**	62,676	36,037	26,639	42.5% (40.5%, 44.5%)	4.1	2.5	1.6	38.00%
	Other		59,258	38,028	21,230	35.8% (33.7%, 37.8%)	3.8	2.5	1.3	35.20%

Table S3: Mortality attributable to socioeconomic inequality by major cause of death, 2003-2018: Females

			Deaths before age 75				Months of life lost before age 75			
Chapter	Major cause of death	ICD10 codes	Observed	Expected	Excess	MASI (95% CI)	Observed	Expected	Excess	YLLI (%)
Cancers	Breast	C50	83,800	80,151	3,649	4.4% (2.5%, 6.2%)	2.8	2.7	0.1	2.00%
	Digestive	C15-26	100,477	84,075	16,402	16.3% (14.7%, 17.9%)	2.5	2.1	0.4	15.40%
	Female genital organs	C51-58	55,661	46,166	9,495	17.1% (14.8%, 19.2%)	1.6	1.3	0.3	18.50%
	Respiratory	C30-39	99,358	52,684	46,674	47.0% (45.7%, 48.3%)	2.3	1.2	1.1	48.60%
	Lymphoid & haematopoietic	C81-96	29,213	26,455	2,758	9.4% (6.4%, 12.5%)	0.8	0.7	0.1	11.00%
	Other		92,676	77,151	15,525	16.8% (15.0%, 18.4%)	2.8	2.5	0.4	12.60%
Circulatory	Stroke	I60-69	49,657	31,369	18,288	36.8% (34.8%, 38.9%)	1.2	0.7	0.5	41.90%
	IHD	I20-25	84,506	39,777	44,729	52.9% (51.6%, 54.2%)	1.8	0.8	1.1	59.00%
	Other		38,833	22,959	15,874	40.9% (38.7%, 43.1%)	1.0	0.5	0.5	46.20%
	Other heart diseases	I30-51	21,462	12,342	9,120	42.5% (39.4%, 45.5%)	0.6	0.4	0.3	43.10%
Neonatal			13,813	9,149	4,664	33.8% (29.1%, 38.5%)	2.3	1.6	0.7	29.20%
Other			81,663	43,942	37,721	46.2% (44.7%, 47.7%)	3.5	1.9	1.6	45.20%
Nervous system		G00-98	38,896	31,560	7,336	18.9% (16.3%, 21.5%)	1.3	1.0	0.3	23.40%
Respiratory	COPD	J40-44	59,351	21,341	38,010	64.0% (62.6%, 65.5%)	1.2	0.4	0.8	68.80%
	Flu & pneumonia	J09-18	23,960	12,094	11,866	49.5% (46.9%, 52.2%)	0.7	0.3	0.3	50.90%
	Other		16,203	9,565	6,638	41.0% (37.5%, 44.3%)	0.4	0.2	0.2	45.50%
Digestive	Liver	K70-76	33,438	17,555	15,883	47.5% (45.3%, 49.8%)	1.4	0.6	0.7	54.20%
	Other		31,157	17,321	13,836	44.4% (41.9%, 46.8%)	0.8	0.4	0.4	46.70%
External	Accidents	**	23,594	14,497	9,097	38.6% (35.4%, 41.6%)	1.3	0.8	0.5	37.50%
	Other		20,541	14,247	6,294	30.6% (26.9%, 34.1%)	1.3	0.9	0.4	31.20%

** X00-59; V00-97; W00-98

4 Map of mortality attributable to socioeconomic inequality by local authority district

An interactive map is available online, which shows the number of deaths attributable to inequality in each local authority, and the underlying causes of the attributable deaths.

Figure S3: Proportion of premature deaths attributable to social inequality (MASI) by local authority, 2003-2018

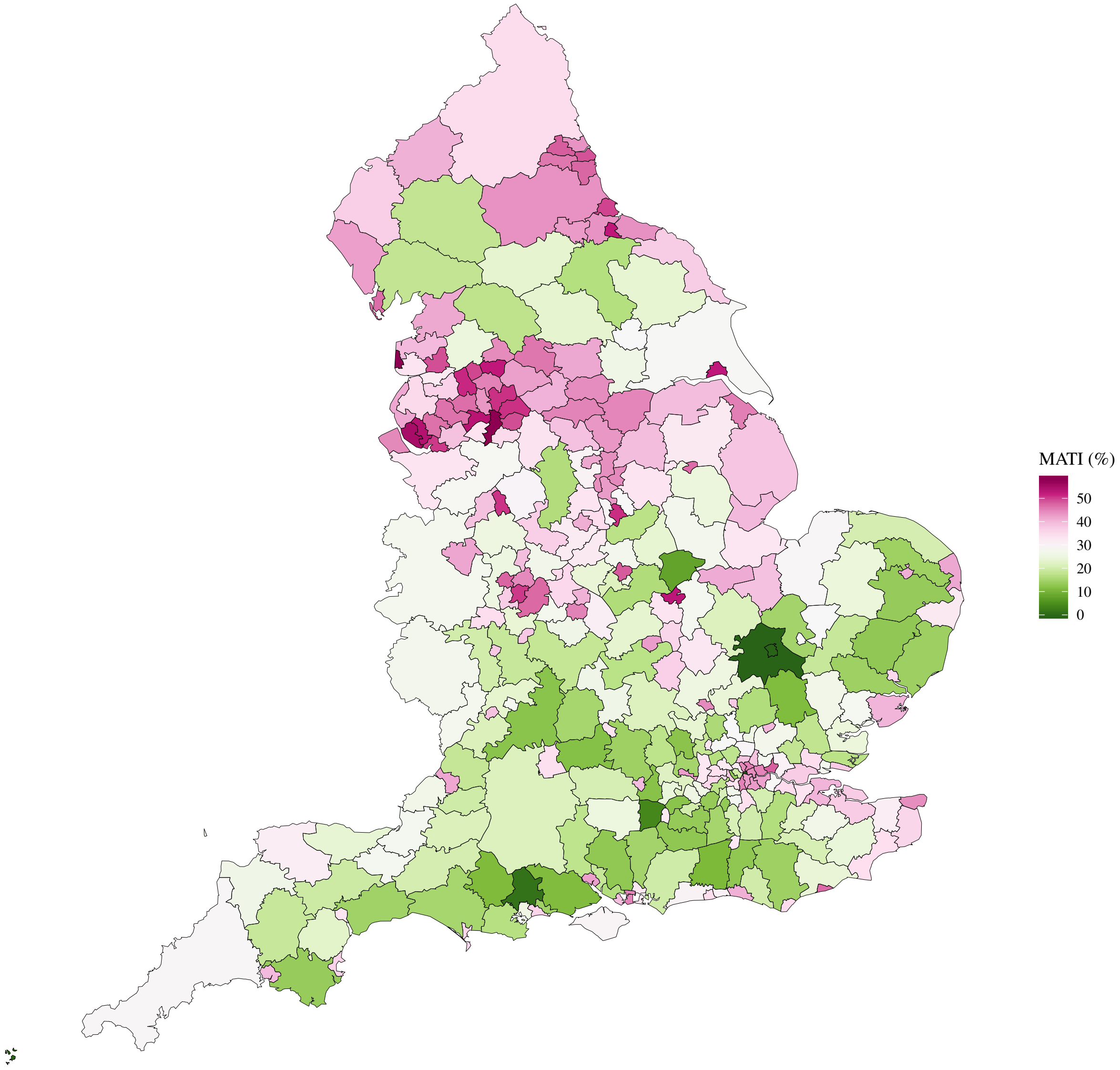

Areas shown are either district councils or unitary authorities, with a total of 326 local authority areas.

5 Mortality attributable to socioeconomic inequality, using different quantiles of deprivation

In our main analysis, we calculated MASI using deciles of the Index of Multiple Deprivation (IMD). Deciles were based on individuals' local neighbourhood (or Lower Super Output Area). Each neighbourhood in England is ranked from 1 (most deprived) to 32,844 (least deprived), with deciles based on these ranks. As a sensitivity analysis, we recalculated MASI using different quantiles of the ranks. For example, the row in the table below showing 2 quantiles represents an analysis where the number of expected deaths is calculated by applying mortality rates in for the less deprived half of the population to the more deprived half. For this analysis we only used data from 2003 to 2017 (i.e. excluding 2018), because we had to use a bespoke population estimate for 2018 and only created this for deciles of deprivation.

Table S4: Mortality attributable to socioeconomic inequality (MASI), recalculated using different quantile of the Index of Multiple Deprivation, 2003-2018

Quantiles	Deaths	Expected Deaths	Attributable deaths	MASI (95% CI)
2	2,323,279	1,802,476	520,803	22.4% (22.3%-22.5%)
3	2,323,279	1,690,740	632,539	27.2% (27.1%-27.4%)
4	2,323,279	1,634,770	688,509	29.6% (29.4%-29.8%)
5	2,323,279	1,597,978	725,301	31.2% (31.0%-31.4%)
6	2,323,279	1,566,496	756,783	32.6% (32.3%-32.8%)
7	2,323,279	1,543,442	779,837	33.6% (33.3%-33.8%)
8	2,323,279	1,524,755	798,524	34.4% (34.1%-34.6%)
9	2,323,279	1,510,846	812,433	35.0% (34.7%-35.3%)
10	2,323,279	1,497,701	825,578	35.5% (35.2%-35.8%)
11	2,323,279	1,484,571	838,708	36.1% (35.8%-36.4%)
12	2,323,279	1,476,213	847,066	36.5% (36.1%-36.8%)
13	2,323,279	1,470,005	853,274	36.7% (36.4%-37.1%)
14	2,323,279	1,464,162	859,117	37.0% (36.6%-37.3%)
15	2,323,279	1,459,394	863,885	37.2% (36.8%-37.6%)
16	2,323,279	1,452,930	870,349	37.5% (37.1%-37.8%)
17	2,323,279	1,448,541	874,738	37.7% (37.3%-38.0%)
18	2,323,279	1,445,088	878,191	37.8% (37.4%-38.2%)
19	2,323,279	1,438,089	885,190	38.1% (37.7%-38.5%)
20	2,323,279	1,432,758	890,521	38.3% (37.9%-38.8%)

6 Change in premature mortality rates between 2003-2006 and 2015-2018, by cause

Table S5: Premature mortality rates per 100,000 between 2003-06 and 2015-2018, adjusted to the European Standard Population 2013, by major cause of death and Index of Multiple Deprivation: Males

			Index of multiple deprivation (1 = most deprived; 10 = least deprived)									
Cause of death		Period	1	2	3	4	5	6	7	8	9	10
Cancers	Breast	2003-2006	0.2	0.2	0.1	0.1	0.2	0.2	0.2	0.2	0.3	0.1
		2015-2018	0.2	0.2	0.2	0.1	0.1	0.2	0.1	0.1	0.1	0.2
		Change	<0.1	<0.1	<0.1	<0.1	-0.1	<0.1	-0.1	<0.1	-0.1	0.1
	Digestive	2003-2006	74.4	66.5	61.6	59.9	55.6	52.9	51.2	51.0	47.1	44.9
		2015-2018	68.9	62.8	56.8	53.0	48.6	47.1	43.8	43.4	41.4	38.3
		Change	-5.6	-3.6	-4.8	-6.9	-7.0	-5.8	-7.4	-7.6	-5.7	-6.6
	Female genital organs	2003-2006	-	-	-	-	-	-	-	-	-	-
		2015-2018	-	-	-	-	-	-	-	-	-	-
		Change	-	-	-	-	-	-	-	-	-	-
	Respiratory	2003-2006	92.8	77.0	63.7	53.8	47.0	42.9	37.7	35.2	33.0	27.1
		2015-2018	73.9	56.6	48.7	39.8	34.4	30.4	26.9	25.7	22.1	18.6
		Change	-18.9	-20.4	-14.9	-14.0	-12.6	-12.5	-10.8	-9.5	-10.8	-8.5
	Lymphoid & haematopoietic	2003-2006	17.1	16.1	15.0	15.8	15.5	15.2	14.9	14.3	15.6	14.7
		2015-2018	13.9	12.9	12.7	12.4	11.5	11.5	11.5	10.9	10.0	10.1
		Change	-3.2	-3.2	-2.3	-3.3	-3.9	-3.6	-3.4	-3.4	-5.6	-4.5
	Other	2003-2006	77.8	74.4	68.3	66.1	63.8	61.6	61.8	57.9	57.6	54.9
		2015-2018	67.1	59.3	56.6	51.9	52.3	48.0	47.9	45.2	44.9	41.3
		Change	-10.7	-15.1	-11.7	-14.2	-11.6	-13.6	-13.9	-12.7	-12.7	-13.6
Circulatory	Stroke	2003-2006	45.8	39.8	33.5	28.2	25.1	24.4	21.7	20.4	19.9	17.2
		2015-2018	26.4	21.2	19.8	15.8	14.0	12.8	11.3	10.5	10.0	8.1
		Change	-19.4	-18.5	-13.7	-12.4	-11.0	-11.6	-10.4	-9.8	-9.9	-9.1
	Ishaemic heart disease	2003-2006	191.7	156.7	136.2	117.8	104.3	93.9	85.4	81.2	74.4	64.3
		2015-2018	116.7	91.8	77.7	65.3	56.8	49.8	45.0	40.7	37.5	31.7
		Change	-75.0	-64.9	-58.5	-52.5	-47.6	-44.1	-40.4	-40.5	-36.9	-32.5
	Other heart disease	2003-2006	20.2	17.1	16.0	14.7	12.4	11.7	10.8	10.0	8.9	8.1
		2015-2018	18.8	14.5	13.4	11.6	10.2	9.5	8.5	7.6	7.1	6.5
		Change	-1.3	-2.6	-2.6	-3.1	-2.2	-2.2	-2.4	-2.5	-1.8	-1.6
	Other	2003-2006	36.3	31.8	27.4	25.6	22.8	20.4	19.1	17.4	16.6	14.4
		2015-2018	26.9	23.3	20.0	17.0	14.3	12.9	11.3	11.2	10.5	9.0
		Change	-9.4	-8.5	-7.4	-8.6	-8.5	-7.5	-7.7	-6.2	-6.1	-5.5
Nervous system		2003-2006	17.6	15.8	15.2	14.5	13.4	13.3	13.3	12.6	12.4	10.8
		2015-2018	17.6	16.2	15.1	14.2	14.3	13.4	13.4	13.4	12.3	11.4
		Change	<0.1	0.5	-0.2	-0.3	0.9	0.2	0.1	0.9	-0.1	0.6
Respiratory	COPD	2003-2006	53.3	44.3	33.6	26.0	23.1	18.9	16.0	14.0	12.1	8.5
		2015-2018	56.3	41.6	32.9	24.6	20.7	17.1	13.6	11.8	10.2	7.3
		Change	2.9	-2.8	-0.7	-1.4	-2.4	-1.8	-2.4	-2.2	-1.9	-1.1
	Flu & pneumonia	2003-2006	24.9	19.6	16.6	12.7	10.7	10.1	8.3	7.7	6.8	6.0
		2015-2018	22.1	16.3	13.9	12.0	9.6	8.2	7.5	6.8	6.5	4.8
		Change	-2.9	-3.3	-2.8	-0.7	-1.1	-1.9	-0.8	-1.0	-0.4	-1.2
	Other	2003-2006	14.8	12.4	11.8	9.6	9.1	8.9	7.6	7.8	7.1	5.6
		2015-2018	12.4	10.3	9.3	7.6	7.1	6.2	5.7	5.8	5.6	4.3
		Change	-2.4	-2.1	-2.5	-2.0	-2.0	-2.6	-1.9	-2.0	-1.6	-1.2
Digestive	Liver	2003-2006	42.2	30.6	25.4	18.3	15.1	13.3	11.4	9.6	9.4	7.5
		2015-2018	37.1	26.7	22.0	17.6	15.8	13.3	11.4	10.9	9.2	7.7
		Change	-5.1	-3.9	-3.4	-0.7	0.7	0.1	-0.1	1.4	-0.1	0.2
	Other	2003-2006	26.6	21.9	18.4	14.8	12.1	11.7	9.8	9.8	8.9	7.1
		2015-2018	21.8	16.4	14.2	11.1	9.6	9.0	7.8	7.3	5.9	5.3
		Change	-4.8	-5.5	-4.2	-3.7	-2.6	-2.7	-2.0	-2.5	-2.9	-1.8
External	Accidents	2003-2006	29.2	23.4	20.7	19.0	17.6	17.0	15.4	14.4	13.7	11.7
		2015-2018	33.3	23.9	19.2	16.9	14.7	13.4	12.1	10.4	9.7	8.3
		Change	4.2	0.5	-1.5	-2.1	-2.9	-3.6	-3.3	-4.1	-4.1	-3.4
	Other	2003-2006	28.8	22.7	19.4	18.1	16.3	14.1	11.9	11.3	10.5	9.9
		2015-2018	21.8	18.1	16.6	15.2	14.2	13.5	12.7	11.3	11.1	9.9
		Change	-7.0	-4.7	-2.9	-2.9	-2.1	-0.6	0.8	0.0	0.6	0.0
Neonatal		2003-2006	7.0	6.0	5.2	4.5	3.9	3.9	3.6	3.0	2.9	2.4
		2015-2018	4.4	3.6	3.0	3.1	2.7	2.5	2.2	2.4	2.4	1.9
		Change	-2.6	-2.5	-2.1	-1.4	-1.2	-1.4	-1.4	-0.6	-0.6	-0.5
Other		2003-2006	64.4	51.4	42.0	36.5	30.6	26.1	24.0	22.6	20.8	16.1
		2015-2018	54.4	45.2	38.3	30.6	26.9	23.7	20.1	19.3	17.0	14.3
		Change	-10.0	-6.2	-3.7	-5.8	-3.6	-2.4	-3.8	-3.4	-3.8	-1.8

Table S6: Premature mortality rates per 100,000 between 2003-06 and 2015-2018, adjusted to the European Standard Population 2013, by major cause of death and Index of Multiple Deprivation: Females

			Index of multiple deprivation (1 = most deprived; 10 = least deprived)									
Chapter	Subgroup	Period	1	2	3	4	5	6	7	8	9	10
Cancers	Breast	2003-2006	27.1	27.3	28.2	27.4	27.3	27.6	27.1	27.4	27.7	26.7
		2015-2018	22.0	20.8	19.3	21.4	20.6	19.5	19.6	19.8	19.5	18.5
		Change	-5.1	-6.5	-8.9	-6.0	-6.7	-8.2	-7.5	-7.5	-8.2	-8.2
	Digestive	2003-2006	37.1	34.0	33.5	31.1	29.5	29.0	28.6	27.3	26.6	25.1
		2015-2018	37.9	33.5	30.9	29.4	27.7	26.7	24.9	24.4	24.1	23.2
		Change	0.8	-0.5	-2.7	-1.7	-1.8	-2.2	-3.7	-2.9	-2.5	-1.9
	Female genital organs	2003-2006	20.3	20.1	18.5	18.6	16.8	17.1	17.0	16.4	16.8	15.2
		2015-2018	17.8	16.5	16.3	15.2	14.0	13.7	13.0	13.5	12.2	11.3
		Change	-2.5	-3.6	-2.2	-3.4	-2.8	-3.4	-4.0	-2.9	-4.6	-3.9
	Respiratory	2003-2006	58.2	44.2	36.9	31.9	26.4	24.1	22.6	20.9	17.8	15.8
		2015-2018	56.5	43.3	36.8	29.6	26.2	23.3	21.1	19.0	17.5	14.1
		Change	-1.7	-0.9	-0.1	-2.3	-0.2	-0.9	-1.5	-1.8	-0.3	-1.7
	Lymphoid & haematopoietic	2003-2006	10.3	10.4	10.3	10.0	9.6	9.3	9.3	9.0	9.2	9.0
		2015-2018	8.1	8.7	7.8	7.1	7.0	6.7	6.4	6.7	6.7	6.4
		Change	-2.1	-1.7	-2.4	-2.9	-2.6	-2.6	-2.9	-2.4	-2.6	-2.6
	Other	2003-2006	37.3	34.8	33.1	30.5	29.8	28.7	28.2	26.6	26.7	24.8
		2015-2018	30.9	28.3	25.5	24.2	23.3	22.6	21.5	20.6	19.9	18.7
		Change	-6.4	-6.5	-7.6	-6.2	-6.4	-6.0	-6.7	-6.1	-6.8	-6.1
Circulatory	Stroke	2003-2006	32.6	26.6	24.2	23.2	19.8	18.5	16.7	16.1	14.9	13.3
		2015-2018	19.6	15.9	14.1	12.4	10.8	9.3	8.9	8.7	8.0	6.6
		Change	-13.0	-10.7	-10.1	-10.8	-9.0	-9.2	-7.8	-7.4	-6.8	-6.6
	Ishaemic heart disease	2003-2006	72.5	56.4	48.1	40.5	33.6	31.3	27.5	25.6	22.7	17.9
		2015-2018	40.6	29.8	26.0	20.5	16.7	14.7	12.5	11.9	9.8	7.6
		Change	-32.0	-26.6	-22.1	-20.0	-16.9	-16.6	-14.9	-13.7	-12.9	-10.2
	Other heart disease	2003-2006	12.2	10.3	8.7	8.2	7.2	6.3	5.9	5.7	5.0	4.2
		2015-2018	10.1	8.7	7.0	6.5	5.7	5.0	4.7	4.1	3.7	3.3
		Change	-2.1	-1.6	-1.7	-1.6	-1.5	-1.3	-1.2	-1.6	-1.3	-0.9
	Other	2003-2006	22.6	20.3	17.3	15.4	13.7	12.0	11.6	11.0	9.8	8.8
		2015-2018	17.0	14.2	12.4	10.5	9.3	8.5	7.7	6.8	6.2	5.2
		Change	-5.6	-6.1	-4.9	-4.9	-4.4	-3.5	-3.9	-4.2	-3.6	-3.6
Nervous system		2003-2006	11.7	11.3	10.3	10.8	10.9	11.1	10.4	9.9	9.7	8.9
		2015-2018	13.4	12.4	12.6	11.9	12.1	11.6	11.0	10.9	10.2	9.0
		Change	1.7	1.1	2.3	1.2	1.3	0.6	0.6	1.0	0.4	0.1
Respiratory	COPD	2003-2006	40.3	31.0	25.2	19.2	16.2	14.3	12.5	10.9	9.2	6.8
		2015-2018	49.2	34.1	25.7	21.1	16.3	13.6	11.4	10.0	8.3	5.6
		Change	8.8	3.1	0.5	1.9	0.1	-0.6	-1.1	-0.9	-0.9	-1.3
	Flu & pneumonia	2003-2006	14.2	12.5	10.4	9.0	7.8	6.8	6.0	5.3	4.7	4.3
		2015-2018	14.4	10.8	9.4	7.7	6.7	6.0	5.3	4.8	4.4	3.4
		Change	0.2	-1.7	-1.0	-1.3	-1.1	-0.8	-0.7	-0.4	-0.3	-1.0
	Other	2003-2006	8.5	8.0	6.1	5.5	4.8	4.7	3.9	4.1	3.9	3.0
		2015-2018	7.0	5.6	4.8	4.2	4.1	3.3	3.0	3.0	2.8	2.2
		Change	-1.5	-2.3	-1.3	-1.3	-0.8	-1.4	-0.9	-1.1	-1.1	-0.9
Digestive	Liver	2003-2006	19.4	14.0	11.4	9.8	8.9	7.5	6.8	6.4	5.5	4.8
		2015-2018	19.1	14.3	11.2	10.1	8.8	7.7	7.0	6.3	5.6	5.1
		Change	-0.3	0.3	-0.1	0.3	-0.1	0.2	0.2	-0.1	0.1	0.3
	Other	2003-2006	18.5	15.4	13.5	11.9	9.8	9.1	8.6	8.3	7.6	6.4
		2015-2018	15.1	12.1	10.5	8.3	7.4	6.6	5.7	5.6	4.9	4.2
		Change	-3.4	-3.3	-3.0	-3.6	-2.5	-2.5	-3.0	-2.7	-2.8	-2.2
External	Accidents	2003-2006	10.5	7.6	7.7	7.0	7.2	6.0	5.6	5.4	5.0	4.6
		2015-2018	13.2	8.9	7.3	6.2	6.3	5.2	4.5	4.3	3.7	3.4
		Change	2.7	1.3	-0.4	-0.8	-1.0	-0.7	-1.1	-1.1	-1.4	-1.2
	Other	2003-2006	9.0	7.3	7.0	5.7	5.9	5.2	4.8	4.6	4.2	3.9
		2015-2018	7.7	6.2	6.2	5.8	5.4	4.5	4.6	4.4	4.2	3.5
		Change	-1.3	-1.1	-0.8	0.1	-0.5	-0.7	-0.2	-0.2	0.0	-0.4
Neonatal		2003-2006	5.7	4.9	4.2	3.8	3.5	3.1	2.9	3.0	2.2	2.2
		2015-2018	3.4	3.0	2.7	2.6	2.2	2.4	1.7	1.8	2.0	1.7
		Change	-2.3	-1.9	-1.4	-1.2	-1.4	-0.7	-1.1	-1.2	-0.2	-0.5
Other		2003-2006	43.3	36.1	32.0	27.5	23.0	21.7	18.9	18.2	16.9	14.4
		2015-2018	40.3	33.6	29.5	24.2	21.8	18.9	16.8	15.6	13.4	11.1
		Change	-3.0	-2.4	-2.5	-3.3	-1.3	-2.7	-2.0	-2.7	-3.5	-3.4

Standardisation was based on the European Standard population 2013 [2]

7 Survival curves by sex and deprivation, estimated by life table modelling

We used mortality rates for entire study period (2003-2018) by single-year-of-age, sex, and deciles of Index of Multiple Deprivation to construct 20 life tables (2 sexes * 10 deciles of deprivation) for cohorts of 100,000. The charts below show the proportion of each cohort that survived at each of age. For men, the proportion surviving to age 75 was 54% for the most deprived decile and 80% for the least deprived decile. For women, the proportions were 69% for the most deprived decile and 86% for the least deprived decile.

Figure S4: Survival curves by sex and deprivation to age 75, England, 2003-2018

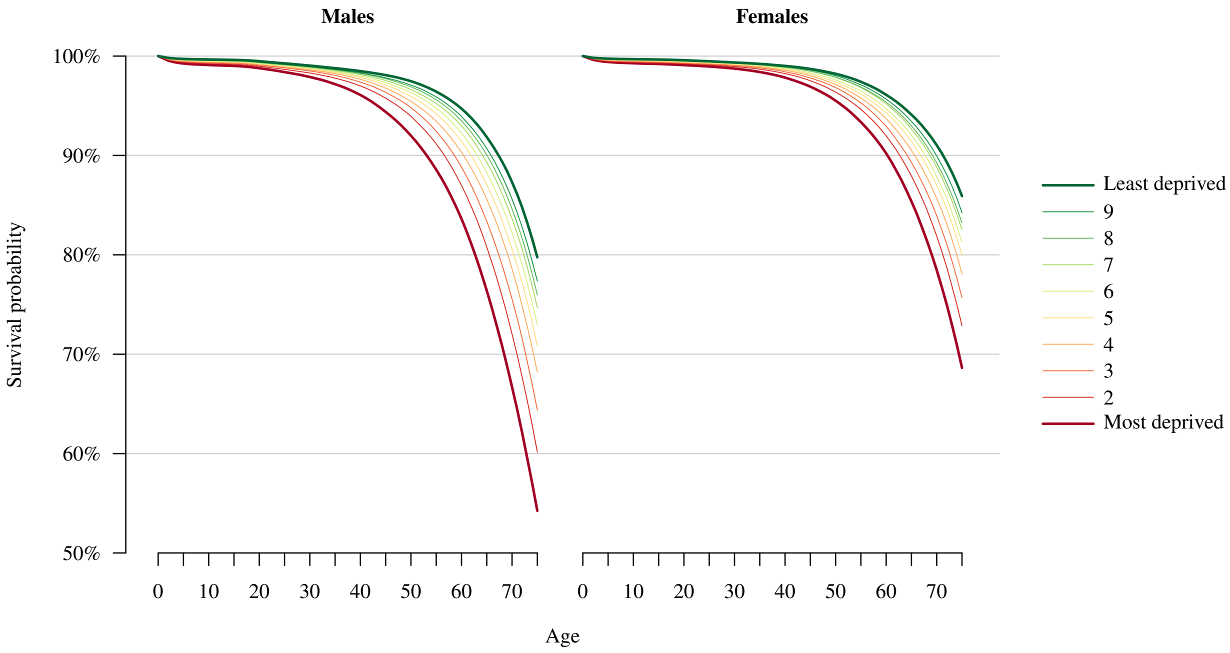

8 References for supplementary information

1. Office for National Statistics. Lower Super Output Area Mid-Year Population Estimates (supporting information). 2017. https://www.ons.gov.uk/peoplepopulationandcommunity/populationandmigration/populationestimates/datasets/lowersuperoutputareamidyearpopulationestimates

2. Pace M, Cayotte E, Agafitei L, et al. Revision of the European Standard Population: report of Eurostat's task force: 2013 edition. 2013. https://publications.europa.eu/en/publication-detail/-/publication/69399636-1113-4a38-9ad1-269c33d0dc01/language-en/format-PDF/source-79847120 (accessed Nov 13, 2018)
